# Clinical spectrum of intrathoracic Castleman disease: a retrospective analysis of 48 cases in a single Chinese hospital

**DOI:** 10.1186/s12890-015-0019-x

**Published:** 2015-04-09

**Authors:** Jin mei Luo, Shan Li, Hui Huang, Jian Cao, Kai Xu, Ya lan Bi, Rui e Feng, Cheng Huang, Ying zhi Qin, Zuo jun Xu, Yi Xiao

**Affiliations:** Department of Respiratory Medicine, Peking Union Medical College Hospital, Chinese Academy of Medical Sciences & Peking Union Medical College, #1 Shuaifuyuan Street, Dongcheng District, Beijing 100730 China; Radiological Department, Peking Union Medical College Hospital, Chinese Academy of Medical Sciences & Peking Union Medical College, #1 Shuaifuyuan Street, Dongcheng District, Beijing 100730 China; Pathological Department, Peking Union Medical College Hospital, Chinese Academy of Medical Sciences & Peking Union Medical College, #1 Shuaifuyuan Street, Dongcheng District, Beijing 100730 China; Department of Thoracic Surgery, Peking Union Medical College Hospital, Chinese Academy of Medical Sciences & Peking Union Medical College, #1 Shuaifuyuan Street, Dongcheng District, Beijing 100730 China

**Keywords:** Castleman disease, Intrathoracic, Unicentric, Multicentric, Clinical characters

## Abstract

**Background:**

Thorax is the common place to develop Castleman disease (CD), but there is no systemic clinical analysis for intrathoracic CD.

**Methods:**

We conducted a retrospective analysis of 48 intrathoracic CD patients with definite pathological diagnosis who were hospitalized between 1992 and 2012 in a Chinese tertiary referral hospital.

**Results:**

The study included 16 cases with unicentric CD (UCD) and 32 cases with multicentric CD (MCD). UCD were younger than MCD (30.5y vs 41.6ys, P < 0.05). MCD were more symptomatic (50% vs 96.9%, P < 0.001) and sicker than UCD, including more fever, hepatomegaly and/or splenomegaly and hypoalbuminemia. All of UCD showed solitary mass in various sites and two of them were complicated by small pleural effusion. In the MCD group, their chest CT showed obvious lymphadenopathy in the hilum and/or mediastinum (100%), diffuse parenchymal lung shadows (43.75%), pleural effusion (40.6%), mass in the mediastinum (6.25%) or hilum (3.12%) and bronchiolitis obliterans (BO) (3.12%). Besides LIP-like images, multiple nodules of different size and sites, patchy, ground-glass opacities and consolidation were showed in their chest CT. Surgery were arranged for all UCD for diagnosis and treatment and all were alive. In MCD group, superficial lymph nodes biopsies (21 cases), surgery biopsy (9 cases) and CT-guided percutaneous lung biopsy (2 cases) were performed. Hyaline vascular (HV) variant were more common in the UCD group (75% vs 37.5%, P < 0.05). In MCD group, 28 cases were prescribed with chemotherapy, one refused to receive therapy and the rest three were arranged for regular follow-up. Among MCD, 18 cases was improved, 7 cases was stable, 4 cases lost follow-up and 3 cases died.

**Conclusions:**

Intrathoracic MCD was more common than UCD in our hospital. MCD was older, more symptomic and sicker than UCD. HV variant were more common in UCD. All of UCD showed mass in various intrathoracic locations and surgery resection was performed for all and all were alive. Mass, pleural effusion, BO and diffuse pulmonary shadows, including LIP-like images, multiple nodules of different size and sites, patchy, GGO and consolidations were showed in our MCD. Most of MCD cases were arranged with chemotherapy and their prognosis were worse than UCD’s.

## Background

Castleman disease (CD) is also known as angiofollicular hyperplasia or giant lymph node hyperplasia. It can be classified as unicentric (unifocal or localized) or multicentric (multifocal or generalized) according to the lesions involved [1-3]. Although CD can occur at any site where lymphoid tissue is normally present, approximately 70% of CD occurred in the thorax [4,5]. Intrathoracic involvement of CD can manifest as a mediastinal and/or hilar mass, pleural mass, tracheal mass, pleural effusion, bronchiolitis obliterans, diffuse parenchymal lung disease, etc. [6-16]. However, most of this data comes from rare case reports or smaller case series. And some of these included cases with the main focus of disease in the neck, supraclavicular and/or axillary nodes [6]. Here we describe the clinical and radiological findings, as well as the outcomes of 48 intrathoracic CD patients in a single Chinese tertiary-referral hospital.

## Methods

### Patients

Using a computer-assisted search for patients who were hospitalized with CD at Peking Union Medical College Hospital from January 1992 to December 2012, we identified 151 CD cases with the confirmed pathological diagnosis. Patients with POEMS syndrome (polyneuropathy, organomegaly, endocrinopathy, M-protein, skin pigmentation) and without complete clinical or radiological data (32 cases) were excluded even if they had the typical pathological features of CD. After review of the medical records, radiologic images and pathological manifestations, 48 intrathoracic CD cases were identified. All of the patients had the main focus of disease inside the chest cage, excluding those with their main involvements in the neck, supraclavicular region, axillary nodes, heart, pericardium and around the aorta. The remaining 71 cases were classified as the extrathoracic CD group.

The following information was recorded for analysis: age, sex, symptoms at presentation, physical examination, and laboratory findings (including serological results, radiologic findings, and pathological results), treatment and outcomes. Patients’ computed tomography (CT) images were downloaded from our hospital’s image data bank. Two radiologists conducted a consensus reading of the CT images. All of the cases with diffuse pulmonary disease were admitted after 1998, so all of these patients had high-resolution chest CT imaging. The biopsies from sections of involved tissues and/or organs were reviewed by two pathologists in our pathological department (Ya lan Bi and Rui e Feng). The pathological diagnosis of CD was established according to Flendrig and Keller’s criteria in all of the enrolled patients [17,18]. The hyaline vascular (HV) variant is characterized by the presence of atrophic germinal centers which are surrounded by expanded mantle zones of small lymphocytes forming concentric rings (“Onion-skinning”), and hyalinized blood vessels penetrating into follicles. For the plasma cell (PC) variant, there is retained lymph node architecture and variable germinal center hyperplasia, with expanded mantle zones and a marked plasmacytosis in the mantle or paracortical regions. Malignant lymphoma, granulomas and histiocytic necrotizing lymphadnitis are excluded. Immunostaining with CD3 and CD20 were performed for both HV and PC variant, but immunostaining with CD38 and CD138 were performed only for PC variant. The mixed variant displays features of both HV and PC variant.

Other definitions in this study included : (1) anemia defined as hemoglobin <110 g/l for female and <120 g/l for male, (2) thrombocytopenia defined as PLT <100 × 10^9^/L, (3) hypoalbuminemia defined as serum albumin < 35 g/l, (4) elevation of immunoglobin G(IgG) defined as serum IgG > 17 g/L, and (5) elevation of lactate dehydrogenase (LDH) defined as serum LDH >270 U/L.

All patient data were followed to September 2014. Data was collected through telephone, letters and cases records. All patients and/or their families provided written informed consent to publish their clinical details. The study was approved by the ethics committee of Peking Union Medical College Hospital.

### Statistical analysis

Data was analyzed using the Statistical Analysis System (SAS) version 8.0 software package. Quantitative variables were described using mean ± SD and categorical data using frequency and percentage in the text and figures. A comparison between two groups was analyzed by the student’s t-test when data was normally distributed. The Kruskal-Wallis test was used when data were not normally distributed. A value of P < 0.05 was considered to be statistically significant.

## Results

Human immunodeficiency virus (HIV) antibody screening test was arranged for 95 CD cases, including 42 intrathoracic cases, and all of them were negative. All cases which were not screened for HIV antibodies were admitted before 2000.

### Demographic characteristics

The intrathoracic CD group consisted of 19 male and 29 female patients with a mean age of 37.9y (range between 13–67 years of age). Among them, 16cases (33.3%) were classified with unicentric CD (UCD) and the remaining 32cases (66.7%) were diagnosed with muticentric CD (MCD). As we know now that there were many differences between the UCD and MCD in clinical, radiological, pathological and prognostic features [1,19,20], we compared the intrathoracic UCD and MCD cases in our hospital (Table 1).

**Table 1:** **Differences between unicentric and multicentric intrathoracic CD cases**

	**UCD**	**MCD**	**P**
**Age (mean, years)**	30.44 ± 8.00	41.56 ± 13.50	0.004
**Gender**			
**Male**	8/50%	11/34.4%	0.2967
**Female**	8/50%	21/65.6%	
**Pathological type**			
**Hyaline vascular**	3/18.75%	16/50%	
**Plasma cell**	12/75%	12/37.5%	0.049
**Mixed**	1/6.25%	4/12.5%	
**Non-symptomatic**	8/50%	1/3.1%	<0.001
**Fever**	0	16/50%	<0.001
**Pleural effusion**	2/12.5%	13/40.6%	0.0475
**Hepatomegaly and/or splenomegaly**	0	16/50%	<0.001
**Anemia**	3/18.8%	15/46.9%	0.0578
**Hemoglobin (g/l)**	133.13 ± 29.70	108.66 ± 26.60	0.0059
**Platelet (×10** ^**9**^ **/l)**			
**>300 × 10** ^**9**^ **/l**	4/25%	12/37.5%	0.6785
**<100 × 10** ^**9**^ **/l**	1/8.3%	2/10%	
**Hypoalbuminia**	4/25%	22/68.8%	0.0041
**Elevation of serum LDH** ^**‡**^	5/30%	5/16.1%	0.6731
**HIV-Ab**			
**Not done**	0	6/18.75%	
**Negative**	16/100%	26/100% (n = 26)	1
**Chemotherapy cases**	0	28/87.5%	<0.001
**Outcomes**			
**Cured**	16/100%	0	
**Improved**	0	18/56.3%	
**Stable**	0	7/21.9%	<0.001
**Died**	0	3/9.4%	
**Lost follow-up**	0	4/12.5%	

^‡^Fifteen UCD cases and 31 MCD cases were detected with serum LDH. LDH: lactate dehydrogenase, HIV: human immunodeficiency virus.

UCD patients were younger than MCD cases, with the mean age of 30.5y and 41.6ys, respectively (t = 3.03, P = 0.004). But there was no difference in the gender distribution between them, with male cases of 50% vs 34.4%, respectively (χ^2^ = 1.09, P = 0.30) (Table 1).

### Clinical and radiological manifestations

The clinical characteristics are summarized in Table 1. Half of the UCD cases had no obvious symptoms when they were admitted to our hospital, but were found to have masses on a routine chest radiographic examination. Thirty-one out of 32 cases with MCD were symptomatic (50% vs 96.9%, χ^2^ = 15.4, P < 0.001). Although fever was the most common symptom for MCD cases (16/50%), there were no patients with UCD presenting with fever (0 vs 50%, χ^2^ = 11.4, P < 0.001). None of the patients with UCD had hepatomegaly and/or splenomegaly, but half of these cases in the MCD group had hepatomegaly and/or splenomegaly (0 vs 50%, χ^2^ = 11.4, P < 0.001).

**Figure 1:**
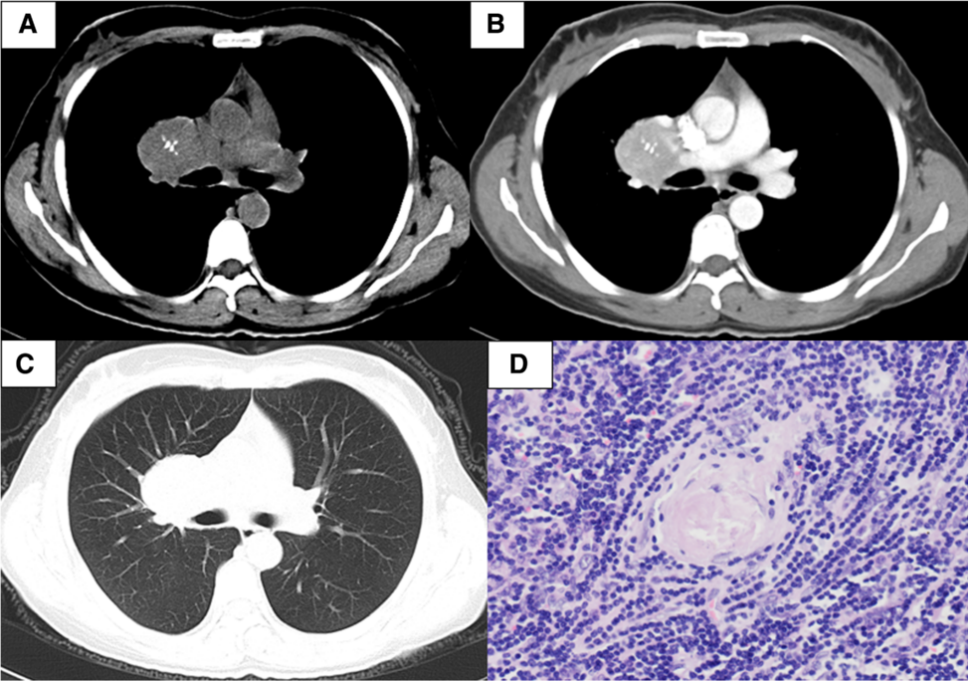
**A 40-year-old woman was diagnosed with unicentric Castleman disease of hyaline vascular variant after thoracic surgery.** She was admitted to our hospital because of slight chest distress for 9 months. Contrasted chest CT showed a well-defined and homogeneous enhanced mass in the right hilum, with coarse calcification **(A-C)**. It was almost normal under the examination of bronscopy and the transbronchial needle aspiration (TBNA) biopsy of the right hilar mass showed chronic inflammation. Whole resection of the mass was performed and prominent diffuse proliferation of lymphoid cells with central hyalinized vascular structures was showed pathologically (**D**, Hematoxylin and eosin, ×200). she was diagnosed with Castleman disease of hyaline vascular variant. Chemotherapy was not suggested. she had been alive without recurrence for 7 years.

In the UCD group, 8 cases complained of at least one of the following symptoms: dyspnea or chest distress (4/25%), dry cough (3/18.75%), backache (1/6.25%) and chest pain (1/625%). Although all cases had a solitary mass in their contrast chest CT, they were distributed in various sites: mediastinum (11 cases/68.75%), lung hilum (3 cases/18.75%), intrapulmonary fissure (1 case/6.25%) and in the lung (1 case/6.25%) (Figure 1). Punctate calcification in the mass was showed in 5 UCD cases. Fourteen cases had been arranged for the chest contrast-enhanced CT, and two cases had been arranged for chest magnetic resonance imaging (MRI). High to moderate enhancement of the mass could be seen in the enhanced CT. The mean size of the mass was 113.56 cm^3^ (ranged from 20 cm^3^ to 336 cm^3^). Two of them were complicated by small pleural effusions.

In the MCD group, 31cases suffered from at least one of the following symptoms: fever (16/50%), cough (15/46.88%), dyspnea (15/46.88%), fatigue (10/31.25%), abdominal distention (6/18.75%), edema (6/18.75%) and chest pain (1/3.12%). Only one patient had no obvious symptoms.

**Figure 2:**
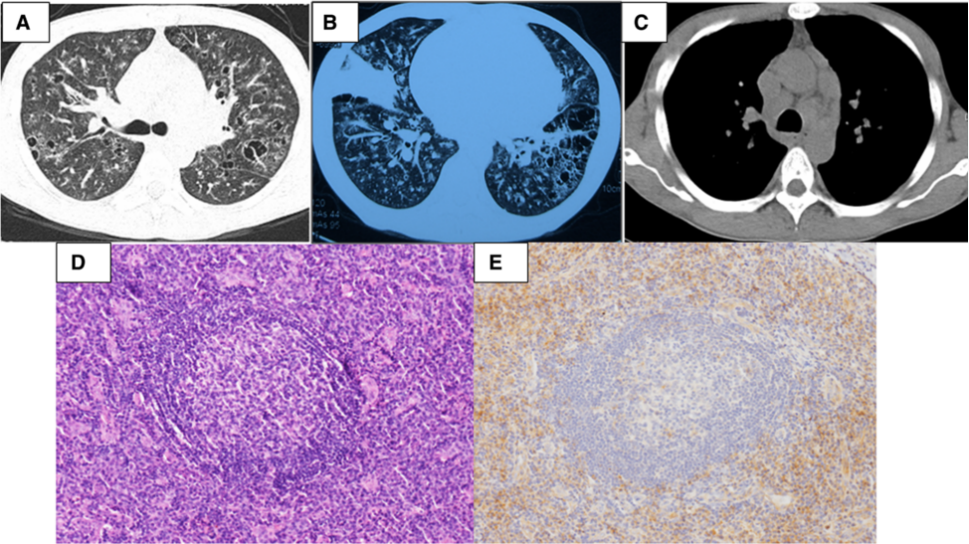
**A 34-year-old man was diagnosed with multicentric Castleman disease of plasma cell variant after 11 years.** He was complained of multiple superficial lymphadenopathy and intermittent low-grade fever for 11 years and exertional dyspnea for 4 months. His local multiple biopsies showed lymph node reactive hyperplasia. Short-term prednisone and repeated kinds of antibiotics were prescribed for him but his symptoms recurred. In our hospital, erythrocyte sedimentation rate (ESR) was 140 mm/h and IgG was 133 g/l, which was polycolonal. His high resolution chest CT showed multiple cysts and nodules, consolidations, diffuse bronchovascular thickening (LIP-like images) and multiple hilar and mediastinal lymphadenopathy **(A-C)**. The fifth biopsy was tried in his left epitrochlear lymph nodes and the pathological photomicrographs showed marked interfollicular infiltration of plasma cells [**D** (Hematoxylin and eosin, ×200) and **E** (CD138 staining, ×200)]. Then he was diagnosed with Castleman disease of plasma cell variant. He and his families refused chemotherapy, but he showed up in the local clinic regularly.

All cases had significant abnormalities on their chest CT (Table 2), including obvious lymphadenopathy in the hilum and/or mediastinum (32/100%), diffuse parenchymal lung disease (14/43.75%), pleural effusion (13/40.6%), mass in the mediastinum (2/6.25%) or hilum (1/3.12%) and bronchiolitis obliterans (BO) (1/3.12%). Thoracentesis was performed in 8 of the cases. Exudative fluid was acquired in 6 of the cases and among them, 5 cases had bilateral effusions. None of the patients had chylous effusions. All of these 6 cases also had high levels of serum IgG. But only one of the cases underwent a pleural biopsy, which showed unspecific chronic inflammation. High-resolution chest CT in 14 of the cases with parenchymal involvement showed lymphocytic interstitial pneumonia (LIP)-like changes in 3 of the cases, multiple nodules of different sizes and sites in 6 of the cases, multiple patchy infiltrates in 6 of the cases, ground-glass opacities (GGO) in 5 of the cases and consolidation in 3 cases (Figure 2).

**Table 2:** **Chest CT manifestations of the enrolled 48 cases**

**Chest CT characters**	**n/%**
**UCDs (n = 16)**	
**Solitary mass (n = 16,100%)**	
**Location**	
**Mediastinum**	11/68.75%
**Lung hilum**	3/18.75%
**Intrapulmonary fissure**	1/6.25%
**Lung**	1/6.25%
**Size**	(113.56 ± 81.31) cm^3^ [(20–336) cm^3^]
**Punctate calcification in the mass**	5/31.25%
**Enhancement in the contrast-enhanced CT (n = 14)** ^**Ф**^	
** Pattern**	
**Homogeneous**	12/85.71%
**Heterogeneous**	2/14.29%
** Degree**	
**High**	13/92.86%
**Moderate**	1/7.14%
** Pleural effusion**	2/14.29%
**MCDs (n = 32)**	
**Lymphadenopathy**	
**Location**	
**Diffuse lymphadenopathy in the hilum and/or mediastinum**	32/100%
**Superficial lymphadenopathy**	20/62.5%
**Cervical LNs**	20/62.5%
**Superclavicular LNs**	4/12.5%
**Axillary LNs**	12/37.5%
**Diffuse parenchymal lung disease**	14/43.75%
**Multiple nodules in different size**	6/18.75%
**Multiple patchy infiltrates**	6/18.75%
**GGO**	5/15.63%
**LIP-like pattern**	3/9.38%
**Consolidation**	3/9.38%
**Bronchiolitis obliterans**	1/3.12%
**Mass**	3/9.38%
**Mediastinal mass**	2/6.25%
**Hilar mass**	1/3.12%
**Pleural effusion**	13/40.6%

^Ф^Two cases with UCDs had been arranged for thoracic MRI instead of the controlled chest CT.

CT: computed tomography, LN: lymph node, GGO: ground-glass opacities, LIP: lymphocytic interstitial pneumonia.

### Laboratory tests

Some of the test results are summarized in Table 1. All 48 cases had complete blood count results. In the UCD group, there were 3 cases (18.8%) who had anemia, with a mean hemoglobin of (133.13 ± 29.70) g/L. In the MCD group, almost half of them (15/46.9%) had anemia and the mean hemoglobin was (108.66 ± 26.60)g/L. It seemed like the MCD patients were prone to suffer from anemia, but the difference was not statistically significant. There was no difference in the platelet counts between the UCD group and the MCD group.

Serum LDH level have been associated with the prognosis of some kinds of lymphoma [21]. We compared the initial serum LDH level between these two groups and it showed no difference. There were 5 cases in both groups with elevated serum LDH (30% vs 16.1%, Z = 0.33, P = 0.67). Hypoalbuminemia was more common in patients with MCD (25% vs 68.8%, Z = 8.22, P = 0.04). As only 3 cases with UCD had been arranged with serum IgG detection, the difference between them could not be compared. In the MCD group, the serum IgG was tested in 27 cases, and the mean serum IgG was 29.5 g/l. Twenty of the cases (74.1%) showed elevated serum IgG, with the highest IgG found to be 133 g/l.

It was reported that kinds of systemic connective tissue disease could complicated by Castleman’s disease [22]. Autoantibodies analysis, including antinuclear antibodies, anti-extractable nuclear antigens (ENA) antibodies, anti-neutrophil cytoplasmic antibodies (ANCA) should be tested for them. As a retrospective study, the results of autoantibodies were acquired in only 4cases with UCD and 30cases with MCD. For these 4 UCD cases, only one had positive ANA with homogeneous pattern at 1:80 titer. For the enrolled 30 MCD cases, 5 cases had positive ANA, with speckled pattern (S), nucleolar (N) or homogeneous pattern (H), ie, S1:160, N1:40, H1:80,H1:320 and H1:320. None of our enrolled 48 cases complicated by a systemic connective tissue disease.

### Pathological features

All patients had a definite pathological diagnosis. Surgery was arranged for all of the 16 cases in the UCD group (Table 3). In the MCD, 21 cases were diagnosed with superficial lymph node biopsies, including cervical (9/28.13%), axillary (6/18.75%) and supratrochlear lymph nodes (1/3.12%). Sixteen cases had at least two superficial lymph node biopsies. Eight cases (25%) were performed with video-assisted thoracic surgery (VATS) for biopsy of mediastinal lymph nodes or lung and one case (3.12%) underwent surgery for biopsy of lymph nodes in the retroperitoneal space. CT-guided percutaneous lung biopsies were arranged for two cases (6.25%).

**Table 3:** **Surgeries for the enrolled UCDs and MCDs**

	**UCD (n = 16)**	**MCD (n = 32)**
**Thoracic surgery**		
**Open thoracic surgery**	16/100%	0
**VATS** ^**ξ**^	1/16.67%	8/25%
**Abdominal surgery**	0	1/3.12%
**Superficial LN biopsy**	0	21/65.6%
**Cervical LN**	0	9/28.13%
**Axillary LN**	0	6/18.75%
**Supratrochlear LN**	0	1/3.12%
**Percutaneous lung biopsy**	0	2/6.25%

VATS: video-assisted thoracic surgery LN: Lymphonode.

^ξ^One case with UCD was performed VATS initially, then he was changed to open thoracic surgery because of severe adhesion in the surgery field.

With respect to our intrathoracic respiratory CD histological type, 24 cases (50%) were classified as the HV variant and 19 cases (39.58%) were diagnosed with thePC variant. The remaining 5 cases (10.42%) were diagnosed with the mixed variant form. HV variants were more common in the UCD group (75% vs 37.5%, χ^2^ = 6.03, P = 0.049) (Table 1).

### Treatment and prognosis

Four cases with MCD were lost to follow-up, and the other cases were available with a mean follow-up time of 75.4 months.

All UCD cases had thoracic surgery for diagnosis and treatment. None of the cases were treated with chemotherapy and all of them were alive.

In MCD group, none of the patients had therapeutic surgery performed, butexploratory surgery for biopsy were arranged for them to acquire pathological diagnosis. Twenty-eight of the 32 cases with MCD were prescribed chemotherapy: CHOP (cyclophosphamide, adriamycin, vincristine and corticosteroid 20cases), COP (cyclophosphamide, vincristine and corticosteroid, 1case), R-CHOP (rituximab and CHOP, 1 case), MP (melphala plus prednisone, 1 case), melphala plus thalidomide and prednisone (1 cases) and corticosteroids plus cyclophosphamide (4 cases). For the remaining 4 MCD cases, 3 of the cases were suggested for regular follow up because of mild symptoms with steady medication for at least 3 years, and the last one refused chemotherapy but complained of superficial lymphadenopathy at multiple sites for 11 years and exertional dyspnea for 2 years (Figure 2).

In MCD group, three cases (9.38%) died: one was complicated by BO and paraneoplastic pemphigus (PNP) and she died of respiratory failure, one had diffuse parenchymal lung disease and suffered from a severe lung infection during the chemotherapy and died, and the other was complicated by myocardial amyloidosis and pulmonary hypertension. All of them were prescribed chemotherapy. Four cases were lost to follow-up after they were discharged from our hospital. Seven of the cases were stable and 18 cases improved after discharge.

## Discussion

Although CD could arise from any site where lymphoid tissue is normally present, the chest was the most common place to develop CD [4-6]. There were several studies which reported thoracic and/or pulmonary CD [4-6,23-25], but most of them were focused on the radiologic features, HIV-associated CD and/or the pulmonary manifestations. Our study is the first of a larger analysis to describe the characteristics of respiratory or intrathoracic CD in a single tertiary-referral hospital.

As HIV antibody screening was not popular in our hospital before the 21 century, HIV tests were not available for 6 MCD cases. But all enrolled cases who had HIV antibody screening had negative results. Those without HIV tests were all admitted before 2000, and HIV infection was not so popular in China before 21 century [26]. So, cases enrolled in our study might be considered as a cohort of non-HIV CD.

Forty percent of the CD in our hospital was located mainly in the thorax, which was less than in previous studies [4,5]. In Kwon’s study, they included the pathologically diagnosed CD involving the thoracic cavity, axilla, chest wall, and the lower neck as thoracic CD [6]. The involved location was not analyzed in Shin’s [19], Dispenzieri’s [1], Robinson’s [27] and Waterston’s [28] studies.

According to the involved lymph nodal station or organ, CD was divided into two clinical subtypes, i.e UCD and MCD. Most studies showed that UCD cases were more prominent than MCD cases [6,19,20]. In Dispenzieri’s study, 53% of cases were UCD [1]. But in Kawabata’s study, MCD was more common than UCD [29]. And Talat et al. reviewed CD cases reported before September 2009, they found that UCD was more common than MCD in HIV-negative CD groups, but all HIV-positive CD cases were MCD [2]. In our study, MCD was more common than UCD. So, the prevalence of different clinical subtypes might vary from different medical centers. And the clinical classification also correlated well with histopathological variants, key clinical features including age, symptoms, treatment strategy and prognosis, and laboratory results, e.g., anemia and serum immunoglobulin levels [1-3,19,29,30], so as our 48 cases with intrathoracic CD. When we compared characteristics between different clinical subtypes of intrathoracic CD in our study, cases in the MCD group were older than in the UCD group, which was consistent with previous studies [19]. But our MCDs was younger than Dossier’s HHV-8 related HIV-negative MCDs, which was suggested to be considered a single clinicopathological entity, regardless of HIV status [31]. As HHV-8 associated tests were not tested for our cases, we could not tell whether this difference was related to HHV-8 infection or not. And there were no gender distribution differences between our UCDs and MCDs.

HV variant cases were more common in our UCD subgroup. Our MCD cases were more symptomic and appeared sicker, including more fever, hepatomegaly and/or splenomegaly and hypoalbuminemia than UCD cases. Our MCD cases showed a lower hemoglobin trend. As there were only 3 UCD cases that had IgG results, we could not compare the IgG difference between the two groups statistically. But 74.1% of the MCD cases in our group who had Ig serum levels checked showed hyperglobulinemia.

Mass in various locations [4-6,32-34], pleural effusion [12,35], bronchiolitis [9,14,36] and diffuse lung shadows [6,15,23,24] had been reported in cases with intrathoracic CD. Although masses could be found in the hilum [6], mediastinum [6,37-39], intercostal spaces [4,5,40], pleura [12], chest wall [32,33], trachea [8,34], lungs [6,41], et al., most of the reported masses were located in the hilum or mediastinum, and there was only rare case report(s) showing masses in other intrathoracic places. There were no more than 20 reported cases showing masses located in the chest wall [32,33]. No more than 10 reported cases with masses in the intercostal spaces [4,5,40], pleura [12], trachea [8,34], lungs [6,41] respectively. Masses could be seen in both of our UCD and MCD cases. All of our intrathoracic UCD showed solitary masses in the mediastinum, hilum, pluera or lungs. As reported in other studies [4,6], surgical resection was performed for our UCD cases and all of them were alive.

Although all of the MCD cases had lymphadenopathy in the hilum and/or mediastinum, only 3 of them showed a significant mass in the hilum and/or mediastinum. There were no cases in our study which had a mass in the chest wall, intercostal space or trachea.

There were no systemic reviews showing CD cases complicated by pleural effusions. Some rare case reports and the analysis of HIV-infected CD cases showed unilateral or bilateral effusions [12,23,24,35,42]. In the case reports the effusion was exudative [12,35] or chylous [42], but there was no effusion tests for HIV-infected CD cases [23,24]. Lesions originating from or involving the chest wall or pleura, impaired drainage of lymphatic fluids because of the compression caused by CD mass or lymphadenopathy, and/or hypoalbuminia could be the causes of pleural effusions in CD cases. Effusion was more common in MCD than in UCD: there were 13 cases (40.6%) with MCD and 2 cases (12.5%) with UCD which were complicated by pleural effusions. Although most of them had bilateral effusions, most of our MCD showed exudative effusions according to Light’s criteria [43]. Without pleural biopsy, we could not explain the causes of effusion.

Follicular bronchiolitis and BO were the reported bronchiolitis in CD cases [9,14,36]. Only Hwangbo et al. reported follicular bronchiolitis in CD cases [14]. But there were some other studies showing the LIP or LIP-like changes in CD cases [6,23,24]. Follicular bronchiolitis and LIP were considered to be on the same spectrum of the disease and the distinction is based on the extent and distribution of the lymphocytic infiltration [14].

There was LIP-like changes in our MCD cases’ chest CT scans. As there was no lung biopsy for these cases, we could not diagnose them as LIP or follicular bronchiolitis. Most of the CD cases complicated with BO had suffered from PNP [9,36], and the pathogenesis of complicated PNP and BO was unknown. But the prognosis of CD associated with PNP and BO was poor. Although most of these cases had complete resection of their masses and/or chemotherapy, most of them died of respiratory failure. Our case with MCD associated PNP and BO died 3 years after thoracic surgery.

Diffuse parenchymal abnormalities had been reviewed by Johkoh et al. [15], Guihot et al. [23,24] and Kwon et al. [6]. In Johkoh’s study [15], they analyzed 12 MCD cases who had diffuse lung disease and 7 cases who underwent surgery or transbronchial lung biopsy and were diagnosed with lymphocytic interstitial pneumonitis (LIP). With similar finding in other chest CT images of cases, LIP seemed to present as the pulmonary manifestations of MCD. Guihot et al. [23,24] reviewed pulmonary involvements in HIV-infected CD. They found diffuse bilateral interstitial pneumonia with bronchovascular thickening, septal thickening and subpleural nodules, GGO, and pulmonary consolidations in these cases, without bronchial dilation and cysts. Most of them had a MCD attack, ie, rapid onset and regression within a few days and with a high frequency of relapse. But besides LIP-like images, multiple nodules in different size and sites, patchy, GGO, and consolidations were shown in our cases’ chest CT scans. On the other hand, some of them had mild symptoms and were stable on medications, so no other therapies were arranged for them. Most of them were stable or improved after therapy.

Our retrospective study had several limitations: First, all enrolled cases had a definite pathological diagnosis, which could result in selection bias. Second, serum Ig analysis was not detected for all enrolled cases, especially for UCD cases. IgG subtype analysis had been performed since 2012 and it was popular after 2013 in our hospital. So, most cases had no initial results of IgG subtype analysis. Third, IgG4-related disease (IgG4-RD) and plasma cell variant CD are especially difficult and often impossible to distinguish based on pathological findings due to their similarities. Serum IL-6 level and HHV-8- related antigen, latency-associated nuclear antigen1, are differential diagnostic factors between IgG4-RD and CD [29]. But all of our cases did not receive tests for HHV-8 and serum interlukin-6 (IL-6) levels, as these items were not carried out in our hospital during that period. Although all of the enrolled cases had no obvious allergic history, which is more common in IgG4-RD patients, some IgG4-RD cases might be enrolled in our cohort. But none of our enrolled cases was revised their diagnosis with IgG4-RD till September 2014.

## Conclusions

MCD was more common than UCD in the intrathoracic CD cases in our hospital. Intrathoracic MCD was older and more symptomic and presented sicker than UCD cases. HV variant cases were more common in our UCD subgroup. All of our UCD cases showed masses in various intrathoracic locations and surgical resection was performed for all of them and all of them were alive. Masses, pleural effusions, BO, and diffuse pulmonary shadows, including LIP-like images, multiple nodules of different sizes and sites, patchy, GGO, and consolidations were shown in our MCD cases. Most of MCD cases were arranged with chemotherapy and their prognosis were worse than UCD’s.

## Abbreviations

CD: Castleman disease
UCD: Unicentric Castleman disease
MCD: Multicentric Castleman disease
CT: Computed tomography
IgG: Immunoglobin G
LDH: Lactate dehydrogenase
HIV: Human immunodeficiency virus
BO: Bronchiolitis obliterans
LIP: Lymphocytic interstitial pneumonia
GGO: Ground-glass opacity
HV: Hyaline vascular
PC: Plasma cell
PNP: Paraneoplastic pemphigus
